# The relationship between natural hydrogen flow rates and production viability

**DOI:** 10.1038/s41598-026-36749-y

**Published:** 2026-01-21

**Authors:** Dieter Franke, Peter Klitzke, Meike Bagge, Ruediger Lutz, Christian Ostertag-Henning, Martin Blumenberg, Christine Thiel, Andreas Bahr

**Affiliations:** Bundesanstalt für Geowissenschaften und Rohstoffe (BGR) , Hannover, Germany

**Keywords:** Natural hydrogen, Techno-economic analysis, Self-replenishing system, Underground hydrogen resources, Environmental sciences, Solid Earth sciences

## Abstract

Despite the widespread occurrence of mostly low concentrations of molecular hydrogen (H₂) in nature, the quantity of commercially recoverable natural hydrogen underground remains uncertain. Two production variants are conceptually considered: a self-replenishing system, in which underground generation balances off-take; and accumulation systems, in which H₂ is trapped underground over long periods and is extracted similarly to conventional natural gas deposits. To assess the potential of natural hydrogen, we compiled and harmonised global data on H₂ flow rates, flux rates, and concentrations from various sources like seeps, springs, mines, and wells. Across different geological settings, observed large natural H₂ flow rates typically fall between 10⁵ and 10⁷ cubic meters per year (m³/yr). When comparing these values to the output of producing natural gas wells and economic viability thresholds for modelled H₂ projects, we find that commercially viable rates must be at least an order of magnitude higher (≥ 10⁷–10⁸ m³/yr). Furthermore, sustained production at high hydrogen purity over two to three decades is generally required for commercial success. Based on this analysis, we argue that economically recoverable natural hydrogen from self-replenishing systems is unlikely.

**Supplementary Information:**

The online version contains supplementary material available at 10.1038/s41598-026-36749-y.

## Introduction

Subsurface molecular hydrogen, i.e. dihydrogen (H_2_) is increasingly discussed as an abundant, low-carbon energy source and for use in “hard-to-abate” industries^1^. The growing interest in this natural hydrogen, variably referred to as geologic, geogenic, white or golden hydrogen is reflected by the increasing number of scientific articles, mainstream media coverage, and start-up companies^2^.

Two conceptual endmembers frame natural hydrogen systems. The replenishing system encompasses continuous rapid generation of natural hydrogen on operational timeframes. The generation rate at least equals the gas escape via e.g. seeps, springs and other biotic or abiotic processes. This concept may imply that natural hydrogen is a renewable resource^2–6^. In contrast, the accumulation system involves the generation of natural hydrogen over longer (i.e. geological) time-scales^1,6,7^. The natural hydrogen accumulates in reservoirs analogous to hydrocarbon systems in sedimentary basins. Thereby, hydrogen charge at least matches in-reservoir losses (biotic and abiotic processes, leakage).

There are more than 30 different processes may produce natural hydrogen gas in the continental crust^8^. Although research continues, to date the two most volumetrically significant processes are thought to be water-rock reactions with iron-bearing minerals and the radiolysis of water from the natural decay of uranium, thorium and potassium^1,9–14^. Due to their comparatively faster reaction rates under favourable temperature–fluid–permeability conditions, water–rock reactions are the only plausible driver of short-term self-replenishment systems, whether natural or anthropogenically stimulated. In contrast, both water–rock reactions and radiolysis can contribute to long-term accumulation systems.

Despite the ubiquitous occurrence of low concentrations of molecular hydrogen in nature, the crucial question for a commercial exploration case is the volume and the reliable determination of this volume. While there is only a single estimate for a hydrogen flow from a well^15^, concentration measurements are widely available but only provide limited volumetric estimates of potential hydrogen accumulations in the subsurface. In contrast, measured surface flux rates and flow rates allow the upscaling of H₂ escape and the calculation of minimum generation rates by integrating en-route losses.

Here, we provide an attempt to estimate the size of potential economic resources of molecular hydrogen underground. We summarise the generation rates, flow rate estimates, and concentration measurements of natural hydrogen obtained from the scientific literature. By comparing these values with data from conventional hydrocarbon gas extraction operations and published values for the economics of hydrogen recovery, we conclude that self-replenishing (renewable), hydrogen systems will unlikely be sufficient to enable an economic recovery of natural hydrogen.

## Results

### Local flow rates of molecular hydrogen

Besides hydrogen purity, production flow rates are the primary cost driver for the economic extraction of natural hydrogen^16,17^. A flow rate indicates the total volume or mass of fluid that moves through a conduit over time. Flux, on the other hand, represents the intensity of the flow, in other words flux is the flow rate per unit area. Concentration measurement alone, defined as the quantity of a constituent divided by the total volume of the mixture, are insufficient for quantitative assessment of gas volumes.

**Fig. 1:**
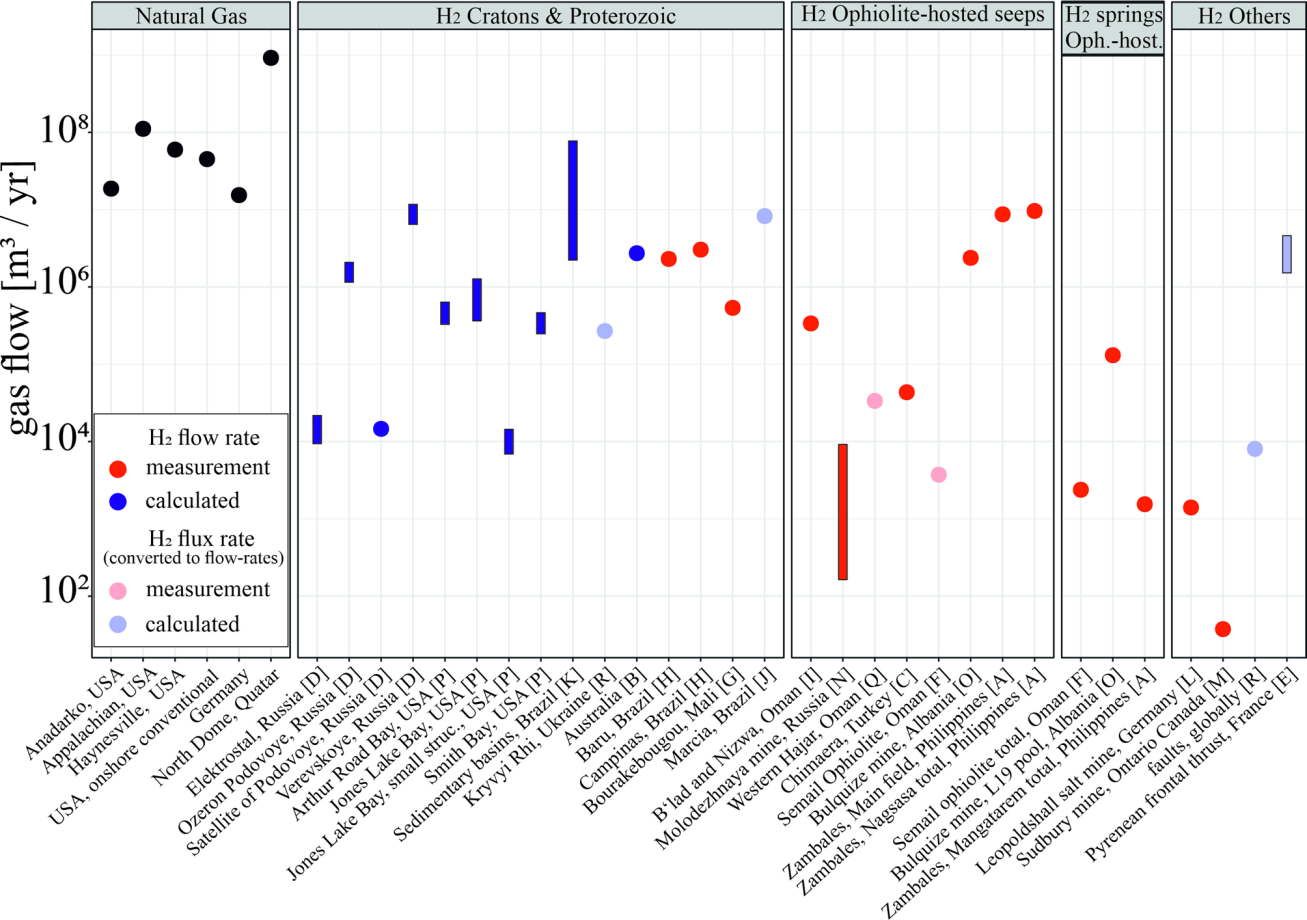
Logarithmic comparison of natural gas and hydrogen flow rates (m³/yr; cubic meters per year). The first column shows flow rates from conventional natural gas wellheads (black circles). Subsequent columns present hydrogen flow data categorized by geological setting. Red circles indicate directly measured flow rates, while blue circles represent calculated flow rates derived from concentration measurements. Bars denote reported ranges. Light red and light blue circles indicate flux-based values that were converted to flow-rate equivalents (see methods). The references for the hydrogen flow rates are as follows (see also Table 1): [A] Aquino et al.^18^, [B] Boreham et al.^19^, [C] Etiope^20^, [D] Larin et al.^21^, [E] Lefeuvre et al.^22^, [F] Leong et al.^23^, [G] Maiga et al.^4^, [H] Moretti et al.^24^, [I] Neal and Stanger^25^, [J] Prinzhofer et al.^3^, [K] Prinzhofer et al.^26^, [L] Rogers^27^, [M] Sherwood et al.^28^, [N] Smith et al.^29^, [O] Truche et al.^30^, [P] Zgonnik et al.^31^, [Q] Zgonnik et al.^32^, [R] Zgonnik^33^. The next chapter describes the natural gas flow rates.

Information on the flow rates of natural hydrogen wellheads is not publicly available. To date, the only documented exception is a water well in Bourakebougou, Mali, which delivered over several years an annual hydrogen flow of 547,500 m^3^/yr (~ 45 tons/yr, based on a 1-day flow-test rate^34^, with a concentration of 98% H_2_^15^.

Figure 1 summarizes measured underground hydrogen flow rates in cubic meters per year (m^3^/yr) on a logarithmic scale. The flow rates shown cover a wide range of settings and sources. The reported values include surface point measurements, estimates integrated over large areas, and the cumulative hydrogen discharge of a mine shaft system, for example. The dataset includes both dedicated measurements of flow rates (red dots) and derived flow rates, e.g. from measurements of concentration gradients and subsequent calculation of the corresponding flow (blue dots). For cases where only flux rates were reported, we estimated flow rates by assuming an average contributing area of 53,000 m^2^ (see methods section). This reference area was used to convert fluxes into flow rates (light red and light blue circles in Fig. 1).

Cratonic settings show comparably high flow rates of around 10^4^ to 10^7^ m³ H₂/yr; however, there are few dedicated flow measurements published. To date, most flow rates have been determined at ophiolites, showing a flow range of mostly 10³ to 10⁷ m³ H₂/yr. Ophiolite-hosted springs and other locations generally have lower flow rates. Overall, the vast majority of measured flow rates plot below 10 million cubic meters hydrogen per year (10^7^ m^3^/yr).

### Natural gas wells productivity

In the absence of publicly available representative hydrogen well test data, the volumetric output of conventional natural gas wells can provide an initial proxy for the required wellhead deliverability for the economic production of natural hydrogen. Established oil and gas industry technologies are likely to be adaptable to natural hydrogen production, although additional, specific challenges need to be addressed.

For conventional natural gas in the U.S., Burnham, et al.^35^ estimate a mean annual production of 28 million m³ per well, recognising that production rates typically decline over time (Fig. 1). This volume equals about 16,660 tons methane (CH_4_), assuming a well methane content of 85 vol.-% on average^35^.

New-well gas drilling productivity per rig is reported by U.S. Energy Information Administration (EIA)^36^. We have chosen the Anadarko, Appalachia, and Haynesville regions as examples because they are predominantly gas provinces, with only limited oil production. In the Anadarko region, the average annual natural gas production from 2007 to 2024 per rig was 18,7 million m^3^ (Fig. 1). In the Appalachian region, about 111 million m^3^/yr were produced per rig, and in the Haynesville region, a single rig produced about 59.7 million m^3^/yr^36^ (Fig. 1). The typical production lifespan of a conventional onshore natural gas well is in the order of 30 years^16,35^. However, some wells may produce for significantly longer periods, particularly in larger deposits.

A second example is Germany, a very mature natural gas province, where production has been declining since decades. This example can be, thus, considered a candidate of the lower-end production category. In 2024 Germany produced 4,100 million cubic meters natural gas from 266 conventional wells^37^. Per production well, this corresponds to 15.41 million m^3^ processed natural gas/yr (Fig. 1). In contrast, in the giant North Dome fossil gas field in Qatar, typical production per well amounts to 919 million m^3^/yr^38^ (Fig. 1). Table 2 summarizes these examples of natural gas production per well.

It is worth noting that hydrogen contains only around a third of the energy of natural gas per unit volume.

### Productivity estimates for underground molecular hydrogen

As Musa, et al.^16^ and Mathur, et al.^17^ pointed out, the economic extraction of molecular hydrogen from the subsurface mainly depends on two critical parameters: The purity of the hydrogen, i.e. the concentration of hydrogen in the produced gas, and the total flow of molecular hydrogen produced from extraction boreholes.

**Fig. 2:**
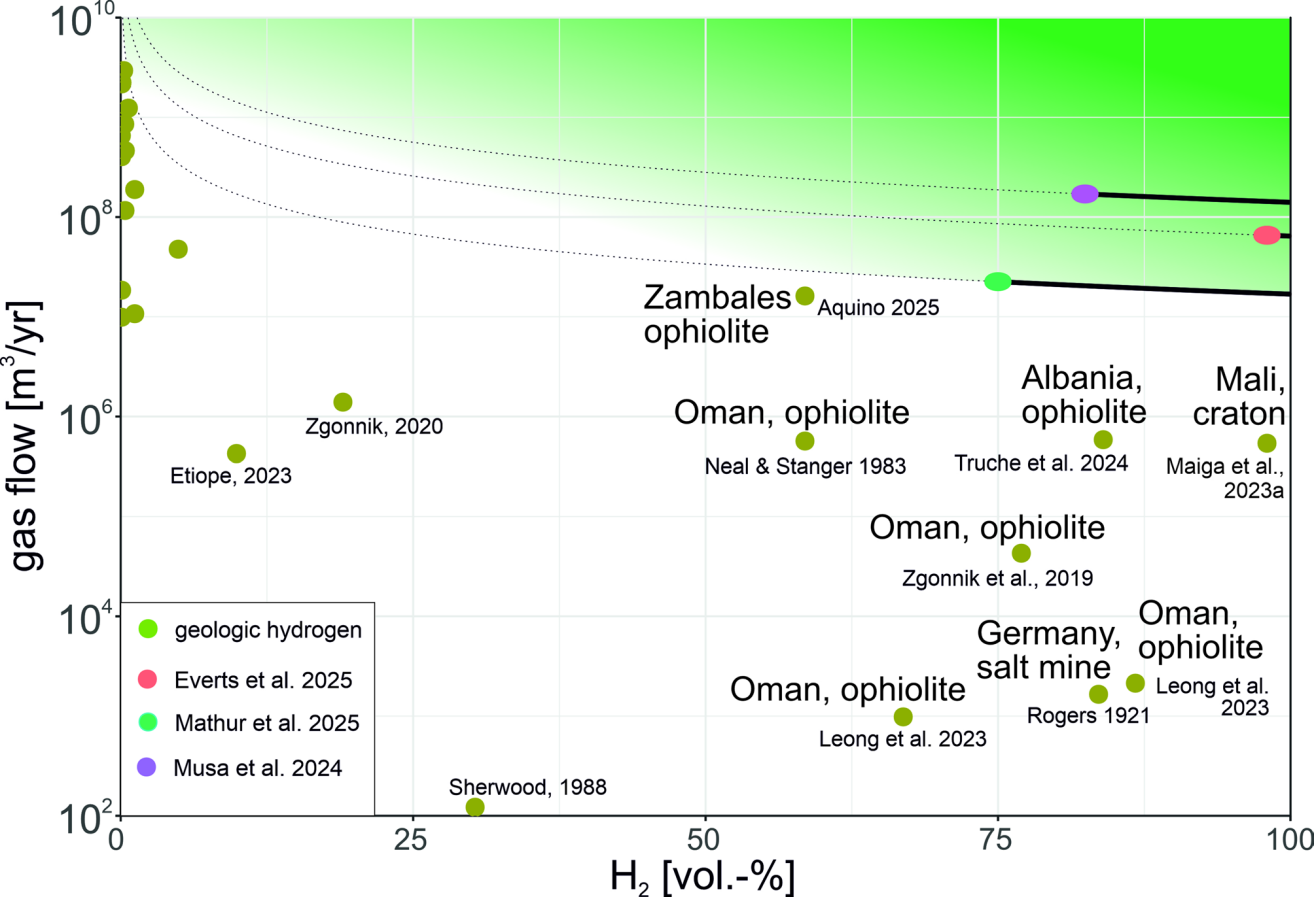
Logarithmic representation comparing measured total gas flow rates (in cubic meters of gas mixture per year) with H_2_ concentration in the gas flow (green circles). Significant values of the total gas throughput with simultaneously high concentrations are labelled. The black curves show base case estimates of economic production from different studies (blue Mathur, et al.^17^, violet Musa, et al.^16^, and red Everts, et al.^34^, extrapolated based on an assumed constant gas composition ratio. The extrapolations to lower H_2_ concentrations are presented as dashed lines. These are highly uncertain, as the effort required for the separation, cleaning and handling of unwanted gas components increases as the H_2_ content decreases.

In their base scenario Musa, et al.^16^ propose 10 hypothetical vertical wells that result in a total production rate of 695 million m^3^/yr, with an assumed hydrogen concentration of 83 mol% (Fig. 2). The base area of the reservoir, assumed to be located in Australia, is set at 650 km^2^. The gas mixture throughput would therefore be 70 million m^3^/yr per well, over a project duration of 30 years, resulting in hydrogen costs of around US$ 2/kg^16^.

A comparable techno-economic analysis by Mathur, et al.^17^ calculated with a much lower wellhead production flowrate of 17 million m^3^/yr and a H_2_ concentration of 75% (Fig. 2) for their base case (resulting in 0,54 US$/kg H_2_). The average lifetime of the well is taken to be 20 years, and these authors calculated 300 days per year production^17^.

Everts, et al.^34^ investigated several hydrogen play-types including focused seepage, coalbed hydrogen and reservoir-trap-seal configuration. They conclude that the latter have the best development potential. A large free hydrogen gas cap in the underground appears to be necessary to realize industrial offtake rates of greater than 586 million m^3^/yr^34^. For the Monzon prospect in Spain, these authors derive a gas volume of 65 million m^3^/yr per well.

The U.S. Department of Energy^39^ has set targets for the stimulated production of natural H_2_. According to these targets, more than 10 million tonnes of H_2_ should be produced per deposit, which would require the stimulation and near-complete exploitation of the H_2_ potential of at least 5–10 km^3^ of peridotite in a geological setting similar to Oman^40^ (assuming a maximum yield of 1 to 2 million tonnes of H_2_/km^3^ for these specific rocks) and H_2_ production of about 351 million m^3^/yr per deposit.

The most advanced natural hydrogen project in Bourakebougou, Mali is often cited as a reference. However, the magnitude must be taken into account. Since 2012, the main borehole has been connected to a pilot plant for power generation, producing 6 kW of electricity for seven years (sometimes 11 years are reported). Nevertheless, this is one location that demonstrates the potential for a cratonic natural hydrogen deposit, albeit one that is still about two orders of magnitude too small for an economic production (see Fig. 2).

When we plot the measured gas flow values against the concentration of H₂ in the respective fluids, we observe that the majority of high gas flow values correspond to a relatively low concentration of H₂. With one exception, those with a considerable H₂ concentration show a gas flow of less than 10^6^ m³/yr. This is a major difference to economic natural gas wells, where the average methane concentration is relatively constant at around 85%. All measured H₂ values are significantly lower than estimates of potential economic occurrence (Fig. 2). Only the Zambales ophiolite in the Philippines shows flow rates that come close to what appears to be necessary for commercial exploitation. In general, examples with high concentrations and flow rates are found in ophiolites (Fig. 2). 

### Global flow rates of molecular hydrogen

The currently known flow rates may be isolated snapshots that fail to capture the most significant hydrogen deposits in the subsurface. To place these observations in a broader context, we compare the measured local flow rates with estimates of global H₂ generation rates. Estimates of global hydrogen formation in the Earth’s crust can differ by orders of magnitude^9,33^, and there has been an upward trend in recent years^9^.

**Fig. 3:**
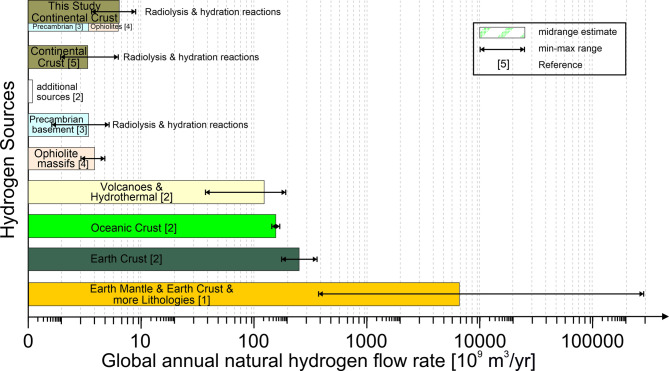
Estimate of global hydrogen flow. The midrange is shown, with the minimum and maximum values indicated. ‘Earth crust’ denotes water-rock reactions with iron-bearing minerals and the radiolysis of water within the crust and minor additional processes. Precambrian basement includes both, radiolysis and hydration reactions. The value suggested by this study is the proposed sum of the annual hydrogen flow from Precambrian basement and ophiolite massifs, i.e. e water-rock reactions with iron-bearing minerals and the radiolysis of water induced by the natural decay of radioactive rock. References: [1] Ellis and Gelman^41^, [2] Zgonnik^33^, [3] Sherwood Lollar, et al.^9^, [4] Zgonnik, et al.^32^, [5] Truche, et al.^10^.

Among the highest estimate of global hydrogen flow is proposed by Ellis and Gelman^41^. These authors estimate the annual flux of hydrogen from the subsurface as the sum of the uncaptured and escaped hydrogen minus the amount consumed by biotic and abiotic processes. As input values, their model uses a H_2_ minimum generation rate of 293 billion m^3^/yr, and a maximum of 293,232 billion m^3^/yr (Fig. 3). The minimum value is based on the global average value by Zgonnik^33^ and the maximum value is primarily based on the assumption of a significant influx of hydrogen from the deep mantle.

In the review article by Zgonnik^33^ the average estimate of the entire annual global production of natural hydrogen is 270 billion m^3^/yr (176–364 · 10^9^ m^3^/yr). However, the aggregation of this value may involve some double counting of hydrogen sources^42^. Furthermore, a significant proportion of this hydrogen is unlikely to be technically or economically recoverable, and therefore must be excluded when considering production potential. Firstly, the generation and release of hydrogen in deep-sea environments is not likely to be exploitable. Of the Earth’s total surface area of 510 million km², the seabed accounts for around 360 million km², i.e. around 71 per cent. Of this ocean surface area, 93% is considered to be the deep-sea floor, covering 335.7 million km². Extracting diffusely outgassing hydrogen at great depths will result in it immediately dissolving in the water and will be extremely difficult, if not impossible. Without these areas, the global potential is reduced by an average of 135 billion m^3^/yr (126–144 · 10^9^ m^3^/yr)^33^. If the estimated 111 ± 83 billion m^3^/yr of further outgassing from volcanoes and hydrothermal systems^33^ is excluded - considered extremely challenging to exploit, and likely unrealistic to extract - then the remaining potential for continental settings reduces to approximately 5,4 ± 2,9 billion m^3^/yr (Fig. 3). Around 3 billion m^3^/yr (2–4 · 10^9^ m^3^/yr^32^ are derived from ophiolite bodies and 2,4 billion m^3^/yr (0,45 − 4,3 · 10^9^ m^3^/yr^33^ from the Precambrian basement.

As the most important processes generating natural hydrogen gas in the continental crust are thought to be water-rock reactions with iron-bearing minerals and the radiolysis of water from the natural decay of uranium, thorium and potassium^1,9–12^, we suggest that 2,580 to 8,680 million m^3^ per year global natural hydrogen generation in the continental crust be used as a baseline. On average the annual natural hydrogen production by Precambrian crust and ophiolites results in about 5,630 million m^3^.

One explanation for the differences in the orders of magnitude for H₂ generation is the consideration of further processes that may contribute, e.g. the generation of hydrogen from kerogen, coal or hydrocarbons^41,43,44^. However, it was precisely the scarcity of H_2_ in the millions of boreholes drilled in sedimentary basins for oil and natural gas that led in the past to the paradigm that there is no abundant natural hydrogen^45^. In addition, whether and to what extent the formation of hydrogen gas in late-stage maturation of sedimentary organic matter is occurring in nature, deep in sedimentary basins at very high pressures in a closed system, or only in pyrolysis experiments at atmospheric pressure and much higher temperatures in an open system is still an ongoing debate^46,47^. We thus do not include this putative generation process for natural hydrogen into our calculations, although we note that research in this area is ongoing.

The largest discrepancy among published global hydrogen generation estimates, however, is caused by whether or not the degassing of the deep Earth’s mantle is considered, i.e. the so-called primordial (primary) hydrogen, which is thought to be stored in the core and mantle of the Earth. We regard the idea of a primordial mantle contribution of H₂ as speculative^1,42^ and suggest to not take this into account unless valid evidence is available (see also supplementary material).

## Discussion

None of the local examples of elevated hydrogen flow discussed in this article were designed to produce H₂ efficiently; they were predominantly discovered by chance. However, despite the reportedly successful initial exploration drilling activities, published data on hydrogen production rates from boreholes is not yet available. If we regard the measured hydrogen at the surface as an indication of larger hydrogen accumulations in the subsurface, then we must hope that the cap rock only released a small fraction of the accumulation only. In any case, comparable natural gas production wells and estimates of the economic viability of natural hydrogen exploration and production suggest a necessary minimum hydrogen flow rate of about 10 million m^3^ per year over two to three decades at the well head.

Few locally measured H_2_ flow rates reach this order of magnitude (Fig. 1) and, those that do show mostly low H_2_-concentrations in the gas mixture (Fig. 2). In addition, it is unclear if the H_2_-flow may hold over decades. Estimates of global onshore flow rates are not promising in terms of supporting much larger flows of hydrogen. Using the estimate by Sherwood Lollar, et al.^9^ and Zgonnik, et al.^32^ of on average 5,630 million m^3^ annual global natural hydrogen generation across the terrestrial part of the Earth’s crust, and the ratio of generated to captured hydrocarbons of 200–300:1^48,49^, this results on average in a magnitude of about 22,5 million m^3^ natural hydrogen that may be annually trapped globally onshore. The fact that the vast majority of samples have H₂ concentrations of less than 0.1%^8^, shows that there are likely to be many small accumulations across the continents. This, in turn, implies that long accumulation times may be required to form hydrogen occurrences of potentially economic relevance.

The question of whether the rate of hydrogen generation could be fast enough to produce it economically from underground sources, without the need for a reservoir, trap and seal is unanswered^50^. Hydrogen generation by radiolysis is certainly too slow. According to estimates by Lin, et al.^51^, with underground isolation, millimolar concentrations of H_2_ are more likely to be generated over periods of millions of years if no consumption takes place. In contrast, hydrogen generation associated with serpentinization may occur on significantly shorter timescales, potentially in the order of years or decades^23,52,53^. However, the maximum replenishment rate for hydrogen systems would need to be measured on a daily level and ideally on an hourly rate.

From the flow rates of molecular hydrogen in the subsurface known to date, it can be deduced that potentially economic deposits, i.e. reserves, can probably only be achieved through enrichment over longer periods of time. Estimates of global hydrogen generation and locally measured flow rates both suggest that a system capable of sustaining industrial offtake rates and replenishing itself rapidly is improbable. Possible hydrogen deposits develop much faster than hydrocarbon deposits, but in return they are less stable and will less likely be preserved over comparably long periods of time. The example of the Bourakebougou hydrogen deposit could be 500 years old, according to a gas proportion model with nitrogen, methane and helium^54^.

Would it be possible to be a little more precise about time scales required for the accumulation of an economic hydrogen deposit? We could use an average underground generation rate of 10 million m³/year (see Fig. 1). Given the aforementioned hydrocarbon trapping efficiency of 200–300:1, it would take approximately 7,500 years to fill a 300-million-m³ H₂ reservoir. The reservoir size assumed here reflects 30 years of exploitation at a rate of 10 million m³ H_2_/yr. However, as 100% recovery is impossible and because of hydrogen losses due to degradation or leakage, a minimum accumulation timescale of approximately 10,000 years appears more realistic. This compares favourably with the 26,000 years operation time of the natural hydrogen system of the Bulqizë ophiolite in Albania^55^. Table 3 provides a brief sensitivity consideration of this calculation and illustrates three different scenarios. Even in an optimistic scenario with high flow rates and efficient trapping, it would take 600 years to fill a reservoir of the required size (Table 3).

Such accumulation time span makes clear that a location with the lowest possible consumption by microbiological activity and abiotic redox reactions is an important basic requirement. Sealing becomes even more important, as hydrogen can leak through minerals and even metals, lowering the change of accumulations after longer times of accumulation. Fault systems as possible reservoirs in crystalline rocks are probably insufficient in volume. This restricts the possible geological settings to subsurface ophiolites, rifts and failed rifts with shallow Moho, and cratonic areas with a larger sediment cover. Neo-tectonic activity might be favourable by enabling fluid flow through young fault systems.

However, the recoverability of an underground deposit depends not only on hydrogen concentration and well head flow rates. Among those are the necessary infrastructure, distance to the customer, qualified personnel, and local acceptance, as well as the reservoir depth and accessibility. One of the largest natural gas deposits in the world, the Russian Shtokman gas field, is estimated to contain around 3 trillion (10^12^) m^3^ of natural gas, has been known for decades and yet is not being developed. The Canadian Svedrup Basin is estimated to contain about 700 billion (10^9^) m^3^ of natural gas, and has remained untouched since the discovery in the 1970s. This shows, in the case of remote gas deposits, transport to the consumer in particular increases the need for correspondingly larger deposits. This means that during ramp-up of production, hydrogen wells likely should produce relatively great volumes to cover costs for the necessary infrastructure. However, the use of natural hydrogen could become more economical through the utilization of associated products such as helium or geothermal energy. As an alternative, the decentralised use of natural hydrogen could be considered, which should also be feasible for smaller deposits.

## Methods

H₂ emissions are reported in the literature (Table 1) in different ways. Some publications provide H₂ flow for a region (in mass/time), while others provide flux (mass/(time · area)). Flux is either measured directly or calculated from concentration measurements (assuming diffusion).

To enable a consistent comparison between studies reporting hydrogen flow rates and those reporting fluxes, we adopted the following procedure. First, we compiled all studies that reported flow rates (mass/time) and calculated the mean flow rate across these sites. We then assumed that studies reporting only fluxes (mass/(time · area)) should, on average, correspond to the same mean total flow rate as derived from the flow-rate dataset.

$$\:{A}_{ref}=\:\frac{{Q}_{mean}}{{F}_{mean}}$$

where.

A_ref_ is the inferred reference area,

Q_mean_ is the mean flow rate from flow-rate studies, and.

F_mean_ is the mean flux from flux-only studies.

This reference area of 0.053 km² was then used to convert all reported fluxes of the individual regions into comparable flow-rate equivalents. We acknowledge that this procedure represents a simplification, as it assumes that flux-only sites are broadly comparable to flow-rate-only sites and that both groups should converge toward the same mean total emission. Nevertheless, this approach provides a practical method for harmonizing heterogeneous data and allows for a first-order comparison of hydrogen release rates across studies.

Flow rates are reported variably in cubic metre H_2_ per cubic metre area (e.g. craton or ophiolite), per day, mol H_2_ per gram transformed ultramafic rock per time, etc. We normalized the values to cubic meters per year, according to conversion factors provided in Table 4.

**Table 1:** Reported hydrogen flow rates.

Reference	H_2_ flow rate [measured vs. calculated]	Geologic setting	Location	H_2_ flow [t/yr]	max. flow [t/yr]	area estimated [y/*n*]	Regional flow [t/y/m^2]	max. regional flow [t/y/m^2]
Aquino, et al.^18^	Measured	Ophiolite-hosted seeps	Philippines, Zambales, Main field	732.00		n		
Aquino, et al.^18^	Measured	Ophiolite-hosted seeps	Philippines, Zambales, Nagsasa, total	808.00		n		
Aquino, et al.^18^	Mmeasured	Ophiolite-hosted springs	Philippines, Zambales, Mangatarem, total	0.13		n		
Boreham, et al.^19^	Calculated	Cratons & Proterozoic	Australia	229.66		N		
Boreham, et al.^19^	Calculated	Cratons & Proterozoic	Australia (< 1 km depth)	134.56	4877.80	N		
Etiope^20^	Measured	Ophiolite-hosted seeps	Turkey, Chimaera	3.65		n		
Larin, et al.^21^	Calculated	Cratons & Proterozoic	Russia, Elekrostal	0.92	1.53	n		
Larin, et al.^21^	Calculated	Cratons & Proterozoic	Russia, Ozero Podovoye	115.11	147.34	n		
Larin, et al.^21^	Calculated	Cratons & Proterozoic	Russia, Satellite of Podovoye	1.23		n		
Larin, et al.^21^	Calculated	Cratons & Proterozoic	Russia, Verevskoye	644.63	828.81	n		
Lefeuvre, et al.^22^	Calculated	Others	France, Pyrenean Frontal Thrust	121.6	260.5	y	2.30E-03	4.93E-03
Leong, et al.^23^	Measured	Ophiolite-hosted seeps	Oman, Samail-Ophiolith	0.25		y	4.75E-06	
Leong, et al.^23^	Measured	Ophiolite-hosted springs	Oman, Haylayn pool	0.16		n		
Leong, et al.^23^	Measured	Ophiolite-hosted springs	Oman, Misfah pool	0.06		n		
Leong, et al.^23^	Measured	Ophiolite-hosted springs	Oman, total	0.20		n		
Maiga, et al.^15^	Measured	Cratons & Proterozoic	Mali, Bourakebougou	45.12		n		
Moretti, et al.^24^	Calculated	Cratons & Proterozoic	Brasil, Baru	193.45		n		
Moretti, et al.^24^	Calculated	Cratons & Proterozoic	Brasil, Campinas	255.50		n		
Neal and Stanger^25^	Meas.	Ophiolite-hosted seeps	Oman, B’lad and Nizwa	28.35		n		
Prinzhofer, et al.^3^	Calculated	Cratons & Proterozoic	Brasil, Marica	555.7		y	1.05E-02	
Prinzhofer, et al.^26^	Calculated		Brasil, sedimentary basin	218.66	5,466.5	n		
Rogers^27^	Measured	Others	Germany, Leopoldshall Salt Mine (Zechstein)	0.12		n		
Sherwood, et al.^28^	Measured	Others	Canada, Sudbury Mine, Ontario	0.003		n		
Smith, et al.^29^	Measured	Others	Russia, Molodezhnaya chromite mine Urals	0.02	0.65	n		
Truche, et al.^30^	Measured	Ophiolite-hosted seeps	Albania, Bulqize chromit mine	200.00		n		
Truche, et al.^30^	Measured	Ophiolite-hosted seeps	Albania, Bulqize, L17 tectonic zone	42.00		n		
Truche, et al.^30^	Measured	Ophiolite-hosted seeps	Albania, Bulqize, L19	158.00		n		
Truche, et al.^30^	Measured	Ophiolite-hosted springs	Albania, Bulqize mine, L19 pool	11.00		n		
Zgonnik, et al.^31^	Calculated	Cratons & Proterozoic	USA, Arthur Road Bay	32.81	44.95	n		
Zgonnik, et al.^31^	Calculated	Cratons & Proterozoic	USA, Jones Lake Bay	36.75	89.91	n		
Zgonnik, et al.^31^	Calculated	Cratons & Proterozoic	USA, Jones Lake Bay, small structure	0.69	1.02	n		
Zgonnik, et al.^31^	Calculated	Cratons & Proterozoic	USA, Smith Bay	24.61	32.81	n		
Zgonnik, et al.^32^	Measured	Ophiolite-hosted seeps	Oman, Western Hajar	2.26		y	4.27E-05	
Zgonnik^33^	Calculated	Ophiolite-hosted seeps	Oman Upper Proterozoic	2.26		y	4.27E-05	
Zgonnik^33^	Calculated	Others	Faults	0.54		y	1.02E-05	
Zgonnik^33^	Calculated	Cratons & Proterozoic	Ukraine, Kryvyi Rih	18.10		y	3.42E-04	

**Table 2:** Examples of natural gas production per well. The data is from Burnham, et al.^35^, U.S. energy information administration (EIA)^36^, energy sector planning and analysis (ESPA)^56^, and Landesamt für Bergbau, Energie und Geologie (LBEG)^37^.

Region	Natural gas production per well [m^3^ per day]	Number of wells	Natural gas production [m^3^ per year]	Natural gas production per well [10^6 m^3^ per year]	Natural gas production per well [tons per year]	Methane (CH_4_) production per well [tons per year]	Methane content in NG [vol.-%]
USA, onshore conventional – mean				28	19,600	16,660	85
Anadarko, USA				18.7	13,092.00	11,127.90	85
Appalachian, USA				111.00	77,694.37	66,040.21	85
Haynesville, USA				59.74	41,818.76	35,545.95	85
Germany	45,318.78	266	4.1 10^9	15.41	10,789.47	10,789.47	99%
North Dome, Qatar	2,518,541.91	208	523,856,718.10	919.27	643,487.46	579,138.71	~ 90%

**Table 3:** Estimating the time required to fill a reservoir of a specified size, based on the determined H₂ flow rates.

	Optimistic	Central	Pessimistic
Underground H_2_ generation rate [m^3^ H_2_/yr]	10^8	10^7	10^6
Trapping efficency	0,005 (1:200)	0,004 (1:250)	0,0033 (1:300)
Reservoir size [m^3^ H_2_]	3 × 10^8^	3 × 10^8^	3 × 10^8^
Time scale to fill the reservoir [yrs]	600	7,500	90,000

**Table 4:** Conversion factors.

H_2_ density (gaseous)	0,085256 kg/m^3^
H_2 (volume-mass-conversion)_	11.729 m^3^/kg
H_2_ calorific value	120 (MJ/kg)
1 Mol H_2_	2.015880 g/mol
CH_4_ density	0.7 kg/m^3^
CH_4_ mass calorific value	55.5 MJ/kg
1 mcf	28,32 m^3^

Figures 1 and 2 are based on the references in Table 1.

### Unit conversion for H_2_ gas mixtures

Different units in the literature were homogenized based on the conversions shown in Table 4.

All conversions are based on the assumption that the perfect gas law is applicable.

$$\:p*V=n*R*T$$

R: gas constant, $$\:8.31445\:J/\left(K*mol\right)$$

p: pressure, 101.325 kPa.

T: temperature, 288.15 K (15 °C).

Mixtures of H_2_ with other gases (O_2_, N_2_, …) are considered.

### Weighted average of the molar mass

In the calculations below the molar mass of H_2_ and a weighted average for the other constituents of the mixture is used.

$$\:{M}_{R}=\frac{\sum\:_{i}^{}{cv}_{i}*{M}_{i}}{\sum\:_{i}^{}{cv}_{i}}$$

$\:{cv}_{i}$: volume percentage (vol%, mol%).

In case of missing information on the associated gases 28 g/mol (M of N_2_) was used as a default.

### Density from molar mass

$$\:\rho\:\left(M\right)=\frac{M*p}{R*T}$$

### Conversion mass% => => vol%

$$\:cv=\frac{cm/{M}_{{H}_{2}}}{\frac{cm}{{M}_{{H}_{2}}}+(100-cm)/{M}_{R}}*100\%$$

$\:cm$: mass percentage (mass%).

### Conversion vol% => mass%

$$\:cm=\frac{cv*{M}_{{H}_{2}}}{cv*{M}_{{H}_{2}}+\left(100-cv\right)*{M}_{R}}*100\%$$

$\:cm$: mass percentage (mass%).

### Volume of gas mixture from H_2_ mass and concentration

$$\:V=\frac{m*w}{\rho\:\left({M}_{{H}_{2}}\right)}+\frac{m*(1-w)}{\rho\:\left({M}_{R}\right)}$$

m: mass of the gas mixture.

$$\:w=\frac{{m}_{i}}{{m}_{total}}=\frac{cm}{100\%}$$: mass fraction.

## Supplementary Information

Below is the link to the electronic supplementary material.

Supplementary Material 1

## Data Availability

All data generated or analysed during this study are included in this published article (and its Supplementary Information files).
