# Supplementary material for: The relationship between natural hydrogen flow rates and production viability
Source: Sci Rep. 2026 Jan 21;16:3036. doi: 10.1038/s41598-026-36749-y (PMC12827248; doi:10.1038/s41598-026-36749-y)
Supplement: Supplementary file 1 — Supplementary Material 1 [file 41598_2026_36749_MOESM1_ESM.pdf]

Supplement (The relationship between natural hydrogen flow rates and production viability)

Evidence for primordial hydrogen degassing from Earth's mantle is lacking.

Mantle outgassing was a popular theory in the middle of the last century, especially in the former Soviet Union (Russian-Ukrainian theory, the Thomas Gold theory or abiotic theory [1]). According to this theory, huge quantities of hydrogen have been stored in the earth's mantle since the earth's origins or water molecules would have split under the pressure and temperature conditions in deep layers of the earth. Originally, the theory was used to explain hydrocarbon deposits, i.e. oil and natural gas deposits. The primordial hydrogen from the earth's mantle should therefore have combined with carbon and formed hydrocarbons [2]. In addition, there is extraterrestrial evidence of traces of hydrogen and methane. It is assumed that meteorites represent the main composition of the material from which the Earth was formed and that hydrogen should therefore also be present.

At the height of the abiotic theory from the 1950s to the 1980s, scientific data for an objective assessment was not yet available. This only became possible with the development of further techniques for analysing the organic components in crude oil and natural gas in the 1980s. Low δ13C values (-5 to -20 $^{0}/_{00}$) characterize methane gas that originates from hydrogen [3], while thermogenic methane shows higher δ13C values (-30 to -50 $^{0}/_{00}$). Accordingly, the abiotic theory can be largely discarded today and is at best speculative from an experimental and observational point of view [4]. Detailed biomarker evidence from oils worldwide as well as geological and experimental considerations clearly speak in favour of a very predominant thermogenic petroleum origin [5]. In a notable investigation of abiotic theory by Apps and van de Kamp [[6], it was concluded that commercial hydrocarbon reservoirs appear to be exclusively biogenic in origin, with the exception of reservoirs possibly associated with serpentinisation. At least for hydrocarbon production (abiotic methane and higher hydrocarbon compounds), which would be based on H_2 outgassing, all abiotic attempts can be considered a failure [7]. Ballentine, et al. [[8] consider it impossible that the Earth's mantle is a source of the hydrogen gas found in the crust or near the surface, since the hydrogen originating from the Earth's mantle is most stable in the form of water at pressures and temperatures at a depth of less than about 90 km.

As the mantle would outgas uniformly, accumulations should be found primarily on deep faults and thinned crust. However, these are precisely the sedimentary basins, the hydrocarbon-rich regions, where at best traces of abiotic methane contribute to the known petroleum deposits. This theory has therefore not played a serious role in hydrocarbon exploration for 40 years. In general, the hydrogen content in most of these conventional hydrocarbon gases is less than 0.1 % [9]. According to Boreham, et al. [[10] natural H_2 deposits in oil and gas fields are modest, with an average H_2 content of about 0.01 mol%. Gases with higher H_2 contents probably have a mixed origin from a deep inorganic source and from the decomposition of organic matter (kerogen) at high maturity levels [10].

The last serious attempt to test the theory of abiotic oil formation took place in the 1980s. Gold succeeded in convincing the Swedish government to drill an ultra-deep well near the granite formations of an old impact crater in northern Sweden. The attempt failed. Only traces of hydrocarbons were found during the drilling [11]. However, the discussion is ongoing [12].

To summarise, traces of abiotic hydrocarbons exist on Earth, but the vast majority of oil and gas deposits are demonstrably biogenic in origin. The likelihood of major accumulations of primordial hydrogen is therefore extremely low at best. As noted by Ballentine, et al. [[8]], it is incumbent on all researchers who assume significant amounts of hydrogen from the Earth's mantle to provide quantitative observations to substantiate such speculation.

Supplementary references

1 Gold, T. & Soter, S. The Deep-Earth-Gas Hypothesis. *Scientific American* **242**, 154-161 doi:https://www.jstor.org/stable/24966351 (1980).

2 Werner, F. *Erdöl – der Wertvollste Rohstoff unserer Zeit*. 132 (Gloria-Verlag AG 1971).

3 Sherwood Lollar, B. *et al.* Evidence for bacterially generated hydrocarbon gas in Canadian shield and fennoscandian shield rocks. *Geochimica et Cosmochimica Acta* **57**, 5073-5085, doi:https://doi.org/10.1016/0016-7037(93)90609-Z (1993).

4 Etiope, G. & Sherwood Lollar, B. ABIOTIC METHANE ON EARTH. *Reviews of Geophysics* **51**, 276-299, doi:10.1002/rog.20011 (2013).

5 Reeves, E. P. & Fiebig, J. Abiotic Synthesis of Methane and Organic Compounds in Earth’s Lithosphere. *Elements* **16**, doi:10.2138/gselements.16.1.25 (2020).

6 Apps, J. A. & van de Kamp, P. C. The future of energy gases. 81-132 (U.S. GEOLOGICAL SURVEY, 1993).

7 Glasby, G. P. Abiogenic Origin of Hydrocarbons: An Historical Overview. *Resource Geology* **56**, 83-96, doi:https://doi.org/10.1111/j.1751-3928.2006.tb00271.x (2006).

8 Ballentine, C. J. *et al.* Natural hydrogen resource accumulation in the continental crust. *Nature Reviews Earth & Environment* **6**, 342-356, doi:10.1038/s43017-025-00670-1 (2025).

9 Smith, N. J. P., Shepherd, T. J., Styles, M. T. & Williams, G. M. in *Petroleum geology : north-west Europe and global perspectives : proceedings of the 6th Petroleum Geology Conference*. (eds A.G. Dore & B.A. Vining) 349-358 (Geological Society of London).

10 Boreham, C. J. *et al.* Hydrogen in Australian natural gas: occurrences, sources and resources. *The APPEA Journal* **61**, 163–191, doi:https://doi.org/10.1071/AJ20044 (2021).

11 Höök, M., Bardi, U., Feng, L. & Pang, X. Development of oil formation theories and their importance for peak oil. *Marine and Petroleum Geology* **27**, 1995-2004, doi:https://doi.org/10.1016/j.marpetgeo.2010.06.005 (2010).

12 Katz, B. J., Mancini, E. A. & Kitchka, A. A. A review and technical summary of the AAPG Hedberg Research Conference on “Origin of petroleum—Biogenic and/or abiogenic and its significance in hydrocarbon exploration and production”. *AAPG Bulletin* **92**, 549-556, doi:10.1306/01210808006 (2008).
